# Deadly Waters as *Naegleria fowleri* Emerges in Brazil - A Call for a One Health Approach to Address Climate Change-Fostered Increasing Threat

**DOI:** 10.1590/0037-8682-0458-2024

**Published:** 2025-12-19

**Authors:** Denise Leal dos Santos, Beni Jequicene Mussengue Chaúque, Luciano Palmeiro Rodrigues, Leila Beltrami Moreira, Leo Sekine, Márcia Santana Fernandes, Marilise Brittes Rott, José Roberto Goldim

**Affiliations:** 1 Hospital de Clínicas de Porto Alegre, Mestrado Profissional em Pesquisa Clínica, Porto Alegre, RS, Brasil.; 2 Laboratório de Pesquisa em Bioética e Ética na Ciência, Porto Alegre, RS, Brasil.; 3 Universidade Federal do Rio Grande do Sul, Porto Alegre, RS, Brasil.; 4 Universidade Rovuma, Center of Studies in Science and Technology, Biology Course, Lichinga, Niassa Branch, Mozambique.; 5 Universidade Federal do Rio Grande do Sul, Faculdade de Medicina, Hospital de Clínicas de Porto Alegre, Porto Alegre, RS , Brasil.

Dear Editor

*Naegleria fowleri*, the etiological agent of primary amoebic meningoencephalitis (PAM), is a thermophilic and ubiquitous protozoan that thrives in environments with temperatures ranging from 30 to 46°C. Remarkably heat-resistant, it can survive temperatures as high as 50-65°C. PAM is an acute, rapidly progressive infectious disease of the central nervous system (CNS) characterized by meningitis-like symptoms and typically resulting in death within 7 days of symptom onset. Common symptoms include high fever, stiff neck, mental confusion, and vomiting. *N. fowleri* enters the body through the nasal cavity where it invades the nasal epithelium and olfactory nerves, passes through the cribriform plate, and proliferates in the olfactory bulb, triggering a severe inflammatory response^1^.

Children and adolescents are most frequently infected, with infection often occurring during recreational activities in contaminated rivers or lakes^2^. Stirring up mud from the bottom of these water bodies can bring amoebae to the surface, increasing the risk of exposure. Additional modes of transmission have been reported worldwide, including nasal irrigation or ablution with tap water, swimming in contaminated swimming pools, playing with water-spraying toys, and using tap water from contaminated reservoirs^3^ ^,^ ^4^.

**FIGURE 1:**
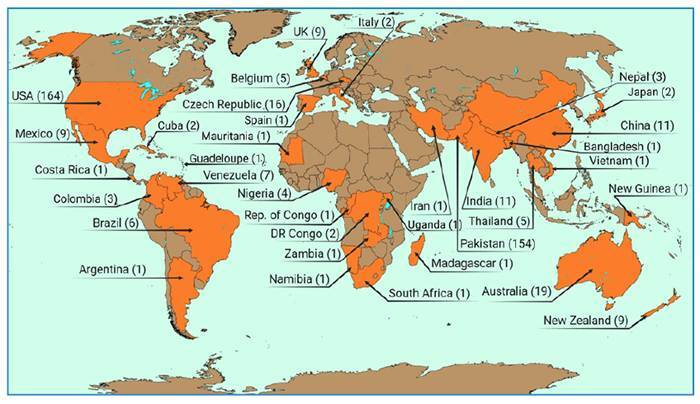
Global Distribution of Primary Amoebic Meningoencephalitis Cases (Data from Nedeem et al.^*7*^ and Rîpă et al.^8^).

PAM has been reported in several regions of the world (Figure 1). According to data from the Centers for Disease Control and Prevention (CDC), 164 cases of *N. fowleri* infection have been documented in the United States between 1962 and 2023, with only four survivors. Cases were initially confined to warm regions including Florida, California, and Texas. However, recent years have reported six cases in the American Midwest, including Minnesota, Kansas, and Indiana^5^. These findings prompt important considerations in response to its increased prevalence fostered by ongoing climate change. Rising temperatures and extreme events such as floods and droughts are global phenomena linked to human activities including gas pollutant emissions, deforestation, and habitat fragmentation. These conditions promote proliferation and spread of pathogens into expanding habitats^6^.

The proliferation of *N. fowleri* in aquatic environments is promoted by rising water temperatures (which can stimulate cyanobacterial blooms, an important food source for the amoeba)^9^ and by the synergistic effects of both factors, which are exacerbated by climate change, further increasing human exposure, particularly during summer when recreational aquatic activities are more frequent^4^. *N. fowleri* is not limited to water; it has also been widely isolated from biofilms, mud, dust, soil, and even desert sand^10^.

In Brazil, five human cases of *N. fowleri* infection were reported between 1972 and 1981^11^. More recently, in December 2024, another case was reported in the State of Ceará, which resulted in the death of a fifteenth-month-old child. The infection likely occurred during bathing in untreated water collected from a reservoir^12^.

According to the Ministry of Health^13^, Brazil recorded 265,644 meningitis cases between 2007 and 2020. Among these, 43,061 cases were attributed to unspecified causes and 2,171 cases had unknown etiology. The latter may include undiagnosed PAM cases. A significant time gap exists between the meningitis cases reported in the 1970s and 80s and the recent case recorded in 2024. This raises concerns regarding potential underdiagnosis of PAM in Brazil, which could stem from lack of clinical suspicion, limited expertise, or the perception of PAM as a rare disease with negligible occurrence. The Brazilian Unified Health System (SUS), one of the largest public health systems in the world, provides universal healthcare to the entire population of the country^14^.

PAM has been reported in several nonhuman mammalian species, including cattle, sheep, black rhinos, and tapirs, in countries such as Algeria and the USA^4^. In Brazil, two fatal cattle infections have been documented in the same municipality (Glorinha, Rio Grande do Sul) on neighboring farms^4^. The presence of PAM in animals has been linked to their contact with contaminated pastures or ponds^15^.

Considering the aspects discussed above, the adoption of an integrative One Health approach is essential to effectively link human, animal, plant, and environmental health. This interconnected framework can enhance the efficacy of our PAM prevention and protection strategies. Given the environmental nature of *N. fowleri* (a ubiquitous organism whose proliferation and human exposure are expected to increase under ongoing climate change) One Health provides a strategic foundation for coordinated interventions. Integrating environmental monitoring, water resource management, epidemiological surveillance, and community education will enable proactive risk assessment and reduce preventive barriers. Microbiological surveillance in recreational waters, coupled with actions to mitigate eutrophication and improve sanitation infrastructure, combats ecological conditions favorable to the pathogen. Concurrent targeted risk communication strategies encourage safer behaviors during periods of elevated water temperature and compromised water quality. By aligning ecological knowledge, public health practices, and climate adaptation efforts, One Health promotes early and effective interventions that address the rapid progression and exceptionally high mortality of PAM, thus reinforcing a sustainable and resilient prevention strategy capable of addressing the evolving epidemiological challenges posed by climate change.
